# Comparative Analysis of Headspace Volatiles of Chinese *Rosa rugosa*

**DOI:** 10.3390/molecules15118390

**Published:** 2010-11-16

**Authors:** Li-Guo Feng, Chen Chen, Li-Xia Sheng, Ping Liu, Jun Tao, Jia-Le Su, Lan-Yong Zhao

**Affiliations:** 1 College of Horticulture and Plant Protection, Yangzhou University, Yangzhou, Jiangsu 225009, China; E-Mails: 29863962@qq.com (C.C.); lxsheng@yzu.edu.cn (L.-X.S.); taojun@yzu.edu.cn (J.T.); 2 College of Forestry, Shandong Agricultural University, Tai’an, Shandong 271018, China; E-Mail: liuping821020@ 126.com (P.L.); 3 Institute of Horticulture, Jiangsu Academy of Agriculture Science, Nanjing, Jiangsu 210014, China; E-Mail: sujl66@yahoo.com.cn (J.-L.S.)

**Keywords:** *Rosa rugosa*, headspacevolatiles, content, gas chromatography–mass spectrometry

## Abstract

The floral headspace compounds of Chinese *Rosa rugosa* germplasms that were isolated by an automated headspace sampler with built-in trap, and followed by gas chromatography-mass spectrometry for identification and quantification. Up to 33 volatile compounds were identified from the 23 rose germplasms, including nine alcohols, five esters, three alkanes, 10 terpenes, three aldehydes, two ketones, and one ether. The main floral components identified were 2-phenylethanol, β-citronellol, ethanol, and *n*-hexane. ‘xizi’, ‘miaofengshan’, ‘xiangciguo’, and ‘tangbai’ contained the highest amounts of 2-phenylethanol at 84.66 μg·g^-1^, β-citronellol at 70.98 μg·g^-1^, ethanol at 83.87 μg·g^-1^, and *n*-hexane at 18.23 μg·g^-1^, respectively. ‘Rongchengyesheng’, ‘tanghong’, ‘xizi’, ‘miaofengshan’, and ‘baizizhi’ could be considered good materials for extracting rose oil and breeding new cultivars.

## 1. Introduction

*Rosa rugosa*, a deciduous shrub of the genus *Rosa*, has prickly stems, pinnately compound leaves, and variously colored, often fragrant flowers. The native range of *R. rugosa* includes Northeastern China [1], Northern Japan [2], the Korean Peninsula [2], and the Russian Far East [3]. In China, wild *R. rugosa* are naturally distributed on the coast and islands of Southern Liaoning Province, Eastern Shandong Province, and Tumen River estuary in Jilin Province, and is classified as an endangered species. In many European countries, it is also used to produce herbal medicines or foodstuffs rich in ascorbic acid, and is extensively used in the breeding of cultivated roses. Due to its hardy and disease-resistant foliage, it has been widely used as a rootstock for grafted roses [4].

*R. rugosa* is economically important in many fields because their petals are a source of rose essential oil, which is also known as “liquid gold”, a very valuable natural raw material, especially in perfumes, cosmetics, aromatherapy, spices, and nutrition [5]. Monoterpene compounds such as citronellol, geraniol, nerol, and their acetate esters are the major headspace compounds of Chinese traditional rose essential oil, and their mass fraction account for 50% to 70% of the total mass fraction in the essential oil [6,7,8,9]. In Bulgaria, France, Turkey, Morocco, and other European countries, rose essential oil is mainly extracted from the flowers of *Rosa damascena*, recently introduced into China; however, compared with the traditional Chinese rose, they display differences in the floral headspace compounds [10,11,12,13,14,15,16,17,18,19,20,21,22,23,24,25].

*R. rugosa* had a long history (over 1,300 years) of cultivation for perfume production in China and numerous varieties and cultivars have been developed mainly through hybridization, sport mutation, and seedling selection. Analytical research on the headspace compounds in rose essential oil has also been carried out for many years using different methods [26], but the content and distribution of the volatile components in the different Chinese *R. rugosa* germplasm resources are still unclear. Thus, identifying specific germplasms that have a high content of monoterpenes and acetate esters is necessary to take full advantage of the rich rose resources in China. In addition, the headspace constituents of rose flowers and essential oil vary and few studies on the headspace compounds in the fresh flowers of *R. rugosa* have been reported [26]. Therefore, this study aimed to evaluate and compare the floral headspace compounds and their content in different germplasms of Chinese *R. rugosa*, as well as to provide useful information regarding rose breeding.

## 2. Results and Discussion

### 2.1. Analysis of floral headspace volatiles in different R. rugosa germplasms

Table 1 lists the code numbers and names of the tested *R. rugosa* germplasms. Table 2 and Table 3 list the volatile components identified from the flowers of *R. rugosa*, as well as their retention indices (RI), and their content in the different samples. In this study, 33 volatile compounds were identified in 23 rose germplasms, namely 9 alcohols, 5 esters, 3 aldehydes, 2 ketones, 1 ether, 10 terpenes, and 3 alkanes. Alcohols, alkanes, and esters were the major headspace compounds because they play dominant roles in the formation of the rose fragrance, whereas the other minor compounds, such as terpenes, aldehydes, ketones and ethers balance and form the specific fragrances of different *R. rugosa* germplasms. The main floral headspace components identified in all *R. rugosa* germplasms were as follows: ethanol, *n*-hexane, 2-phenylethanol and β-citronellol, the latter two compounds are the main constituents of 23 *R. rugosa* germplasms because they had the greatest aromatic intensity.

**Table 1:** Code numbers and names of the tested *R. rugosa* germplasms.

Code number	Name	Code number	Name
R1	‘zhongkeerhao’	R13	‘xiangciguo’
R2	‘zixiadiancui’	R14	‘tangfen’
R3	‘ciguo’	R15	‘mupingyesheng’
R4	‘tangbai’	R16	‘rongchengyesheng’
R5	‘ziyingbinfen’	R17	‘tanghong’
R6	‘zhongkeyihao’	R18	‘xizi’
R7	‘jinxiujiangshan’	R19	‘miaofengshan’
R8	‘zifurong’	R20	‘zizhi’
R9	‘Chongbanhong’	R21	‘dahongzizhi’
R10	‘tangzi’	R22	‘fenzizhi’
R11	‘zilongwochi’	R23	‘baizizhi’
R12	‘zhuzishuanghui’		

The total alcohol varied content among the 23 germplasms, with ‘xizi’ having the highest content at 252.01 μg·g^-1^, followed by ‘tanghong’ at 204.96 μg·g^-1^, whereas ‘zhongkeerhao’ had the lowest content at 5.38 μg·g^-1^. The most abundant alcohol compounds were 2-phenylethanol, β-citronellol, ethanol, and *cis*-geraniol, which were already reported as the major components in the fresh flowers and essential oil of Chinese *R. rugosa* [20,27].

The average 2-phenylethanol content among the 23 *R. rugosa* germplasms was 27.19 μg·g^-1^, with ‘xizi’ showing the highest content at 84.66 μg·g^-1^, followed by ‘rongchengyesheng’, ‘tanghong’, and ‘baizizhi’, which all exceeded 60 μg·g^-1^, whereas ‘zixiadiancui’ had the lowest content at 0.31 μg·g^-1^. The average β-citronellol content for all the tested samples was 25.80 μg·g^-1^, with the maximum level of 70.98 μg·g^-1^ being measured in the fresh flowers of ‘miaofengshan’, whereas ‘tangfen’, ‘tanghong’, and ‘xizi’ also had significant levels at 52.50, 67.56, and 67.93 μg·g^-1^, respectively, and the lowest level was noted in ‘zhongkeerhao’ at only 2.33 μg·g^-1^.

The ethanol levels in the different *R. rugosa* flowers were highly significant, with an average content of 25.97 μg·g^-1^; ‘xiangciguo’ had the maximum content at 83.87 μg·g^-1^, followed by ‘tangfen’ at 82.72 μg·g^-1^ whereas ‘zhongkeerhao’ had the lowest content at 0.67 μg·g^-1^. *cis*-Geraniol, another major component of Chinese *R. rugosa*, was not detected in ‘jinxiujiangshan’, ‘zilongwochi’, ‘zizhi’, or ‘fenzizhi’. Its average content in the other 19 samples was 3.96 μg·g^-1^, with ‘tanghong’ displaying the maximum content at 17.53 μg·g^-1^, whereas ‘tangfen’, ‘rongchengyesheng’, and ‘xizi’ also had significant contents at 8.35, 8.12, and 9.44 μg·g^-1^, respectively. The lowest content was seen in ‘zhongkeerhao’ at only 0.04 μg·g^-1^.

Esters were also found in 22 of the 23 samples (the exception being ‘rongchengyesheng’), with an average content of 8.13 μg·g^-1^. ‘Miaofengshan’ contained a high total amount of esters at 34.39 μg·g^-1^. The total ester contents in ‘baizizhi’, ‘fenzizhi’, ‘tanghong’, and ‘dahongzizhi’ were also relatively high at 26.2, 20.16, 17.23, and 17.02 μg·g^-1^, respectively. The other samples did not contain more than 9 μg·g^-1^. Citronellol acetate was the most abundant ester in the samples, at an average of 6.50 μg·g^-1^. ‘Miaofengshan’ had the maximum content at 30.85 μg·g^-1^ and ‘fenzizhi’, ‘baizizhi’, ‘dahongzizhi’, and ‘tanghong’ also had significant levels at 18.88, 13.69, 12.52, and 12.22 μg·g^-1^, respectively. Nerol acetate had been described as most important to rose flavor [15], according to our results, it was found in only nine samples, and its concentrations were highest in ‘tanghong’, ‘dahongzizhi’, and ‘miaofengshan’ at 4.21, 3.69, and 2.39 μg·g^-1^, respectively.

Three alkanes were identified in the flowers of 23 *R. rugosa* germplasms, with *n*-hexane being the most abundant at an average content of 8.90 μg·g^-1^ whereas ‘tangbai’ and ‘mupingyesheng’ had the maximum and lowest content at 18.23 and 1.46 μg·g^-1^, respectively. In addition, *trans*-rose oxide, an important constituent of the rose-specific aroma, was detected in 8 *R. rugosa* germplasms at an average of 1.60 μg·g^-1^. 

In conclusion, ‘tanghong’, ‘xizi’, and ‘miaofengshan’ had the highest amounts of 2-phenylethanol, β-citronellol, *cis*-geraniol, citronellol acetate, and nerol acetate, making them good materials for extracting rose oil and breeding new varieties. 

### 2.2. Analysis of floral headspace volatiles in some specific R. rugosa germplasms

‘Mupingyesheng’ and ‘rongchengyesheng’, two wild germplasms introduced from the coast and islands of Eastern Shandong Province of China, are classified as an endangered species [1]. Consequently, protecting and rationally using these precious wild germplasm resources is imperative. As shown in Table 2 and Table 3, 13 volatile compounds, mainly alcohols, esters, aldehydes, ketones, terpenes and alkanes, were identified in ‘mupingyesheng’, whereas ethers were not detected. The main headspace compounds were β-citronellol, 2-phenylethanol, *cis*-geraniol, and ethanol, with β-citronellol as the most abundant at 11.14 μg·g^-1^, compared with the other components.

Twelve compounds, including alcohols, aldehydes, ketones, terpenes, and alkanes, but not esters and ethers, were identified in ‘rongchengyesheng’, among which 2-phenylethanol, β-citronellol, *cis*-geraniol and ethanol were the major headspace compounds. The 2-phenylethanol content was the highest at 73.11 μg·g^-1^. The total amount of headspace substances in ‘rongchengyesheng’ reached 124.88 μg·g^-1^, 3.76 times that seen in ‘mupingyesheng’, and the alcohols were at levels up to 118.4 μg·g^-1^ or four times the level in ‘mupingyesheng’. Consequently, ‘rongchengyesheng’ can be considered a potential high value germplasm.

Until now, the differences in headspace compounds between *R. rugosa* cultivar and its mutants have not been reported. ‘Zizhi’ had an important industrial and ornamental value with excellent features such as amaranthine flowers, amaranthine branches, and multi-seasonal flowering. ‘Dahongzizhi’, ‘fenzizhi’, and ‘baizizhi’ are sport mutations of ‘zizhi’, and their flowers are red, pink, and white, respectively. According to our results, 11, 10, seven, and 14 volatile compounds were identified in the flowers of ‘zizhi’, ‘dahongzizhi’, ‘fenzizhi’, and ‘baizizhi’, respectively. Only alcohols, esters, and alkanes were detected in ‘zizhi’, with alcohols being the major components. In contrast, only alcohols, esters, terpenes, and alkanes were detected in ‘dahongzizhi’, ‘fenzizhi’, and ‘baizizhi’, with alcohols and esters as their main components.

**Table 2:** Headspace constituents and their content of the different *R. rugosa* germplasms (μg·g^-1^).

Constituent	RI^a^	R1	R2	R3	R4	R5	R6	R7	R8	R9	R10	R11	R12	R13	R14	R15	R16	R17	R18	R19	R20	R21	R22	R23
Ethanol	485	0.67	1.71	1.82	15.74	19.57	37.55	45.25	0.77	2.78	2.85	27.91	46.32	83.87	82.72	2.71	5.64	55.18	85.30	4.89	61.72	2.17	5.25	4.87
3-Methylpentane	583	0.32	0.49	0.45	0.65	0.38	0.47	0.42	0.13	0.27	0.48	0.39	0.14	-	0.32	-	-	-	-	-	0.29	-	-	1.77
*n*-Hexane	600	10.18	15.59	13.70	18.23	11.74	14.41	10.25	5.93	7.71	14.08	11.30	8.63	13.68	11.70	1.46	3.03	2.09	4.86	4.72	11.31	1.89	3.07	5.03
3-Methylcyclopentene	615	-	0.22	-	-	0.14	0.17	-	-	-	-	-	-	-	-	-	-	0.25	-	-	-	-	-	-
Methylcyclopentane	634	0.50	0.79	0.71	-	0.58	0.72	0.55	0.31	0.35	0.69	0.55	0.42	-	0.51	0.28	0.71	-	-	0.30	0.55	-	-	-
Isopentyl alcohol	736	-	-	0.08	0.43	0.09	0.28	0.20	-	-	-	-	-	0.55	-	-	-	0.55	-	-	-	-	-	0.67
*cis*-3-Hexen-1-ol	844	-	-	-	0.09	-	0.13	-	-	-	-	-	-	-	-	-	-	-	-	-	2.02	-	-	1.93
*n*-Hexanol	858	-	-	0.21	0.32	0.24	0.30	0.38		-	-	0.42	0.29	0.61	0.83	-	-	0.54	1.10	-	0.61	-	-	0.54
2-Heptanone	872	0.08	0.40	0.12	0.25	0.15	0.19	0.12	-	0.11	-	-	-	-	-	0.21	0.31	0.62	1.47	0.38	-	0.29	-	-
5-Methyl-2-hexanol	892	0.12	-	0.14	0.21	0.12	0.29	0.27	-	-	-	-	0.33	0.67	-	0.30	0.75	0.97	2.43	-	-	-	-	-
α-Pinene	926	0.32	0.44	0.49	0.35	0.40	0.19	-	0.75	1.54	-	-	-	2.58	2.12	-	-	1.97	1.48	2.21	-		-	-
β-Pinene	985	-	-	-	-	-	-	-	-	-	-	-	-	-	-	-	0.50	-	-	-	-	1.15	-	-
β-Myrcene	990	-	0.29	0.31	0.90	0.44	0.35	-	0.13	0.18	-	-	-	-	0.64	0.59	-	1.66	0.66	1.00	-	-	0.98	0.86
4-Hexen-1-ol, acetate	992	-	0.25	-	0.14	-	-	-	0.17	-	-	0.39	-	-	-	0.48	-	-	-	-	2.02	0.81	-	3.23
*n*-Hexyl acetate	997	0.04	0.52	0.20	0.31	-	-	-	0.36	0.17	0.42	0.58	0.25	0.53	0.58	0.19	-	0.80	1.03	-	-	-	-	1.40
benzyl alcohol	1002	-	1.28	-	1.37	-	-	-	-	-	-	-	-	-	-	-	0.68	1.22	1.15	-	-	-	-	-
*D*-Limonene	1013	-	0.19	0.15	0.50	0.31	0.28	-	-	-	-	-	-	-	0.44	-	1.41	0.62	-	-	-	-	-	-
3-Carene	1017	-	-	0.08	0.14	0.06	-	-	-	-	0.15	-	-	-	-	-	-	-	-	-	-	-	-	-
Ocimene	1035	-	-	-	0.90	-	-	-	-	-	-	-	-	-	-	-	-	1.26	-	-	-	-	-	-
*trans*-β-Ocimene	1043	-	0.21	-	-	-	-	-	-	-	-	-	-	-	-	-	-	0.52	-	-	-	-	-	-
2-Ethylidene-6-methyl-3,5-heptadienal	1066	-	-	0.32	0.23	0.12	0.16	0.48	0.29	-	0.45	-	1.40	1.56	1.89	-	-	-	0.94	1.47	-	-	-	-
2-Phenylethanol	1080	2.22	0.31	8.92	21.7	25.51	37.33	32.26	5.27	4.28	11.30	13.89	10.03	39.09	31.02	9.91	73.11	61.41	84.66	31.53	28.45	7.10	20.21	65.85
*trans*-Rose oxide	1111	-	-	0.19	-	-	-	0.08	0.52	-	3.94	-	1.50	5.19	0.24	-	-	-	1.11	-	-	-	-	-
β-Citronellal	1134	-	0.05	-	-	0.08	0.12	0.55	-	-	-	-	0.07	-	0.52	-	-	0.92	0.37	0.35	-	-	-	-
β-Citronellol	1229	2.33	16.43	20.01	18.4	24.62	26.76	19.89	5.36	8.20	21.47	6.72	34.27	31.56	52.50	11.14	30.10	67.56	67.93	70.98	6.85	10.68	18.20	21.55
β-Phenethyl acetate	1233	0.33	-	0.80	-	-	-	0.37	1.90	0.65	1.07	1.82	-	1.61	-	-	-	-	-	1.15	0.35	-	1.28	4.92
α-Citral	1237	-	0.21	-	0.42	-	0.59	0.41	-	-	-	-	-	-	0.59	0.26	0.52	1.73	0.94	-	-	-	-	-
*cis*-Geraniol	1258	0.04	2.24	0.68	4.77	3.79	4.34	-	0.31	0.38	0.22	-	2.73	0.75	8.35	5.17	8.12	17.53	9.44	3.95	-	0.64	-	1.88
2-Undecanone	1279	-	-	-	0.16	-	-	-	-	-	-	-	-	-	-	-	-	0.17	-	-	-	-	-	-
Citronellol acetate	1335	0.71	3.15	4.80	2.32	3.66	1.82	1.39	0.92	6.01	4.79	0.46	5.30	3.23	1.43	0.54	-	12.22	8.29	30.85	5.96	12.52	18.88	13.69
Nerol acetate	1345	-	0.35	0.63	0.54	0.23	-	-	0.45	0.46	-	-	-	-	-	-	-	4.21	-	2.39	-	3.69	-	-
Aromadendrene	1458	-	0.37	-	-	-	-	-	-	-	-	-	-	-	-	-	-	-	-	-	-	-	-	-
α-Farnesene	1518	-	-	0.64	0.10	-	0.28	0.66	-	-	0.87	-	0.75	-	-	-	-	-	0.94	-	-	-	-	-

^a^ The Kovat's retention indices on apolar column Rtx-1; - Not detected.

**Table 3:** Aromatic categories and total headspace volatiles of 23 *R. rugosa* germplasms (μg·g^-1^).

Compound	R1	R2	R3	R4	R5	R6	R7	R8	R9	R10	R11	R12	R13	R14	R15	R16	R17	R18	R19	R20	R21	R22	R23
Alkanes	11.00	16.87	14.86	18.88	12.7	15.6	11.22	6.37	8.33	15.25	12.24	9.19	13.68	12.53	1.74	3.74	2.09	4.86	5.02	12.15	1.89	3.07	6.80
Alcohols	5.38	21.97	31.86	63.03	73.94	106.98	98.25	11.71	15.64	35.84	48.94	93.97	157.1	175.42	29.23	118.40	204.96	252.01	111.35	99.65	20.59	43.66	97.29
Esters	1.08	4.27	6.43	3.31	3.89	1.82	1.76	3.8	7.29	6.28	3.25	5.55	5.37	2.01	1.21	-	17.23	9.32	34.39	8.33	17.02	20.16	23.24
Terpenes	0.32	1.72	1.67	2.89	1.35	1.27	0.66	0.88	1.72	1.02	-	0.75	2.58	3.20	0.59	1.91	6.28	3.08	3.21	-	1.15	0.98	0.86
Aldehydes	-	0.26	0.32	0.65	0.2	0.87	1.44	0.29	-	0.45	-	1.47	1.56	3.00	0.26	0.52	2.65	2.25	1.82	-	-	-	-
Ketones	0.08	0.49	0.12	0.41	0.15	0.19	0.12	-	0.11	-	-	-	-	-	0.21	0.31	0.79	1.47	0.38	-	0.29	-	-
Ethers	-	-	0.19	-	-	-	0.08	0.52	-	3.94	-	1.50	5.19	0.24	-	-	-	1.11	-	-	-	-	-
Total headspace volatiles	17.86	45.49	55.45	89.45	92.23	126.73	113.53	23.57	33.09	62.78	64.43	112.43	185.48	196.4	33.24	124.88	234.00	274.1	156.17	120.13	40.94	67.87	128.19

The main floral headspace compounds identified in ‘zizhi’, ‘dahongzizhi’, ‘fenzizhi’, and ‘baizizhi’ were ethanol, *n*-hexane, 2-phenylethanol, β-citronellol, and citronellol acetate; ethanol was the most abundant component in ‘zizhi’ at 61.72 μg·g^-1^, and 2-phenylethanol, *n*-hexane, β-citronellol, and citronellol acetate were also present at significant amounts at 28.45, 11.31, 6.85, and 5.96 μg·g^-1^, respectively. The ethanol and *n*-hexane content of ‘zizhi’ were significantly higher than in the mutants, but β-citronellol and citronellol acetate were more abundant in the mutations. Except for alcohols and esters, the content of the other headspace compounds in ‘dahongzizhi’ were lower; among them was citronellol acetate, which was the highest at 12.52 μg·g^-1^. The ethanol, *n*-hexane, and 2-phenylethanol levels in ‘dahongzizhi’ were significantly lower than in the other three germplasms; β-citronellol and citronellol acetate were also lower than those in both ‘fenzizhi’ and ‘baizizhi’, however, nerol acetate was present in ‘dahongzizhi’ but was completely absent in ‘zizhi’, ‘fenzizhi’, and ‘baizizhi’.The 2-phenylethanol, citronellol acetate, and β-citronellol in ‘fenzizhi’ were at 20.21, 18.88, and 18.20 μg·g^‑1^, respectively. Citronellol acetate, in particular, was significantly higher than in the other three germplasms. ‘Baizizhi’ had the largest number of detected compounds among the four germplasms, with its total amount of headspace substances reaching 128.19 μg·g^-1^, of which 2-phenyl-ethanol and β-citronellol were remarkably higher than in the other three germplasms. Therefore, ‘baizizhi’ may be considered a precious resource for extracting rose oil because of its high amounts of 2-phenylethanol, β-citronellol and citronellol acetate.

## 3. Experimental

### 3.1. Plant material

A total of 23 *R. rugosa* germplasms were used in this study, including two wild germplasms and 21 cultivars. Table 1 shows the code numbers and names of the 23 germplasms that were collected from the *R. rugosa* germplasm repository of Shandong Agricultural University, China.

### 3.2. The headspace volatiles extraction

Three to five robust rose branches from each germplasm that had the most flowers were cut under water and immediately placed inside a vase with clean water, and were then quickly transported to the laboratory for testing. Five full freshly opened flowers were selected. Their petals were gently plucked and mixed. Up to 2 g of petals were quickly placed in 15 mL sample vials, internal standard (3-nonanone, 10 μL, 0.8 μg·μL^-1^) were added, and the vials were then sealed rapidly with septa (PTFE-butyl synthetic rubber). Extraction and concentration of the floral headspace volatiles from the fresh flowers were performed using a TurboMatrix 40 Trap (Automated headspace sampler with built-in trap, PerkinElmer, USA). The extraction conditions was as follows: The heating temperature was at 35 °C for 30 min, the temperature of the sampling needle and transmission line was 80 °C, the sample vial head pressure was 15 Pa, and trap was maintained for 5 min.

### 3.3. Gas chromatography with mass spectrometry (GC-MS) analysis

GC-MS was carried out using a GCMS-QP2010 Plus (Shimadzu Corporation, Japan). The mass spectral ionization temperature was set to 200 °C. The electron energy was 70 eV. Mass spectra were obtained by automatic scanning at m/z 45-450 amu.

The flow rate of the helium carrier gas on Restek Rtx-1 (30 m × 0.25 mm I.D., 0.25 μm film, Restek Corporation, USA) was 1.03 mL/min. Then, 1.0 μL of the sample was injected in the splitless mode at an injector temperature of 200 °C. The column temperature was programmed as follows: The initial temperature was maintained at 40 °C for 2 min, and then increased from 40 °C to 130 °C at 4 °C/min, maintained at 130 °C for 1 min, and finally increased to 230 °C at a rate of 7 °C/min, which was maintained for 4 min.

### 3.4. Qualitative and quantitative analysis

Statistical analysis was done using the Xcalibur software. Qualitative analysis was performed as follows: spectrometric data were compared with those obtained from the NIST HP59943C original library mass spectra (Hewlett-Packard) and the Wiley library, combined with the manual resolution of mass spectra and confirmed by comparing the Kovat's retention indices and relative reports from the literature. Only results identified with positive and negative matching values of more than 800 (maximum is 1,000) were shown in this article. 

Quantitative analysis was done with 3-nonanone (10 μL at 0.8 μg·μL^-1^; Sigma Ltd. Co., St. Louis, MO, USA) as the internal standard. The selected ion monitoring technique was used for quantitative analysis of the headspace compounds, whereas the response factor method combined with the internal standard method was used to quantify these compounds. The mass fraction was calculated using the formula given below: 

Content of each component (μg·g^-1^) = [Peak area of each component/peak area of internal standard × Concentration of internal standard (μg·μL^-1^) × Volume of internal standard]/ Sample weight (g)

## 4. Conclusions

This study revealed a high level of floral headspace compound polymorphism in Chinese *R. rugosa* plants. Alcohols, alkanes, and esters were the major headspace compounds, whereas the main floral components that were identified in the 23 germplasms were ethanol, *n*-hexane, 2-phenylethanol, and *β*-citronellol. ‘Tanghong’, ‘xizi’, and ‘miaofengshan’ had high amounts of 2-phenylethanol, β-citronellol, *cis*-geraniol, citronellol acetate, and nerol acetate. ‘Rongchengyesheng’, a precious wild germplasm, is a potentially high-value germplasm because of its high aromatic intensity. After comparing the headspace compounds in a specific cultivar and its sport mutations, ‘baizizhi’ was verified as a significant source of rose oil due to its high concentrations of citronellol acetate and β-phenethyl acetate. In conclusion, ‘rongchengyesheng’, ‘tanghong’, ‘xizi’, ‘miaofengshan’, and ‘baizizhi’ are good materials for extracting rose oil and breeding new cultivars.
